# Optimization of Synthesis of the Amino Lipid ECO for Effective Delivery of Nucleic Acids

**DOI:** 10.3390/ph14101016

**Published:** 2021-10-02

**Authors:** Andrew L. Schilb, Josef H. Scheidt, Amita M. Vaidya, Zhanhu Sun, Da Sun, Sangjoon Lee, Zheng-Rong Lu

**Affiliations:** 1 Department of Biomedical Engineering, School of Engineering, Case Western Reserve University, Cleveland, OH 44106, USA; als241@case.edu (A.L.S.); jhs192@case.edu (J.H.S.); amv74@case.edu (A.M.V.); sunzhanhu@hotmail.com (Z.S.); dxs564@case.edu (D.S.); sxl1755@case.edu (S.L.); 2 Case Comprehensive Cancer Center, Case Western Reserve University, Cleveland, OH 44106, USA

**Keywords:** gene therapy, ECO, miRNA, siRNA, plasmid, RNA interference, synthesis

## Abstract

Nucleic acids are promising for a variety of therapies, such as cancer therapy and the gene therapy of genetic disorders. The therapeutic efficacy of nucleic acids is reliant on the ability of their efficient delivery to the cytosol of the target cells. Amino lipids have been developed to aid in the cytosolic delivery of nucleic acids. This work reports a new and efficient synthetic pathway for the lipid carrier, (1-aminoethyl) iminobis [*N*-(oleicylcysteinyl-1-amino-ethyl)propionamide] (ECO). The previous synthesis of the ECO was inefficient and presented poor product quality control. A solution-phase synthesis of the ECO was explored, and each intermediate product was characterized with better quality control. The ECO was synthesized with a relatively high yield and high purity. The formulations of the ECO nanoparticles were made with siRNA, miRNA, or plasmid DNA, and characterized. The transfection efficiency of the nanoparticles was evaluated in vitro over a range of N/P ratios. The nanoparticles were consistent in size with previous formulations and had primarily a positive zeta potential. The ECO/siLuc nanoparticles resulted in potent luciferase silencing with minimal cytotoxicity. The ECO/miR-200c nanoparticles mediated the efficient delivery of miR-200c into the target cells. The ECO/pCMV-GFP nanoparticles resulted in substantial GFP expression upon transfection. These results demonstrate that the solution-phase synthetic pathway produced pure ECO for the efficient intracellular delivery of nucleic acids without size limitation.

## 1. Introduction

Gene therapy is a powerful tool that allows for the modification, editing, or regulation of gene expression to correct genetic dysfunctions and pathologies. Multiple avenues, such as gene replacement, gene editing, or RNA interference (RNAi), are available for gene therapy by normalizing, correcting, or regulating genetic functions. These therapies require the delivery of therapeutic mRNAs, DNA, CRISPR/Cas, siRNAs, oligonucleotides, or miRNAs in the cytoplasm of target cells for curative outcomes [1]. For instance, in the case of RNAi-mediated oncogenic silencing for cancer therapy, the cytosolic delivery of therapeutic RNA, including siRNAs, is imperative for their facile access to the RNA-inducing silencing complex (RISC)-bound complementary mRNA targets to enable targeted oncogene knockdown with minimal side-effects [2,3]. These considerations warrant overcoming multiple barriers for the delivery of nucleic acids, such as nuclease degradation, innate immune response, enhancing target cell uptake, endosomal escape, and cytosolic release in disease-specific tissues for in vivo clinical use [4,5,6,7,8,9].

Decades of research has gone into addressing these barriers, and various gene delivery vehicles, in particular lipid nanoparticles, have shown promise for gene delivery. Lipid nanoparticles offer unique opportunities for the efficient delivery of nucleic acids by overcoming the practical challenges with several advantageous features, including good biocompatibility, the ability to deliver genetic cargo of all sizes, and limiting immune recognition [10,11,12,13,14,15]. Several lipid nucleic acid nanoparticles have undergone successful clinical trials such as Patisiran/ONPATTRO in targeting transthyretin for hereditary transthyretin amyloidosis, and the recent LNPs mRNA vaccines against COVID-19 [2,16].

We have developed a simple and smart amino lipid 1-aminoethylimino[bis(*N*-oleoylcysteinyl-aminoethyl)propionamide] (ECO) to serve as a multifunctional pH-sensitive carrier; it previously showed success in the delivery of genetic materials such as siRNA, miRNA, and plasmid DNA for treating various diseases [17,18,19,20]. The ECO allows for the formation of nanoparticles through the electrostatic head group with the negatively charged nucleic acids and the hydrophobic condensation of the oleic acid tails. Additionally, the ECO possesses two thiol groups that can be used to modify the nanoparticles with targeting agents through maleimide chemistry to increase specificity for targeted delivery. Various targeting agents can be conjugated via a PEG spacer via a hydrazone linker to enhance endosomal escape [17]. The unreacted thiols are capable of crosslinking through disulfide bond formation by autoxidation in order to enhance nanoparticle stability. Once formulated, these nanoparticles offer long-term storage while preserving nanoparticle stability and functionality [21]. The resulting nanoparticles were shown to efficiently deliver genetic materials via pH-sensitive amphiphilic endosomal escape and reductive cytosolic release, or the PERC effect [22]. The effectiveness of the ECO for in vivo delivery of nucleic acids was demonstrated in multiple gene therapy studies for cancers and retinal disorders [17,18,19,20,23].

The ECO were previously synthesized through standard solid-phase chemistry [23]. However, it is a challenge to achieve proper quality control in each step of the synthesis, resulting in low yield of the final product. In this work, we sought to optimize the synthesis of the ECO using liquid-phase synthetic chemistry to achieve better quality control and the desirable scalability for further clinical development and applications. The intermediates and final product were characterized with ^1^H-NMR, MALDI-TOF, and HPLC. The ECO from the liquid phase synthesis was tested to form nanoparticles with siRNA, miRNA, and plasmid DNA, respectively, at varying N/P ratios. The ECO/nucleic acid nanoparticles were characterized by dynamic light scattering and zeta potential. The in vitro transfection and biological functions of the ECO/nucleic acid nanoparticles were characterized in different cell lines.

## 2. Results and Discussion

### 2.1. Synthesis of ECO

**Scheme 1:**
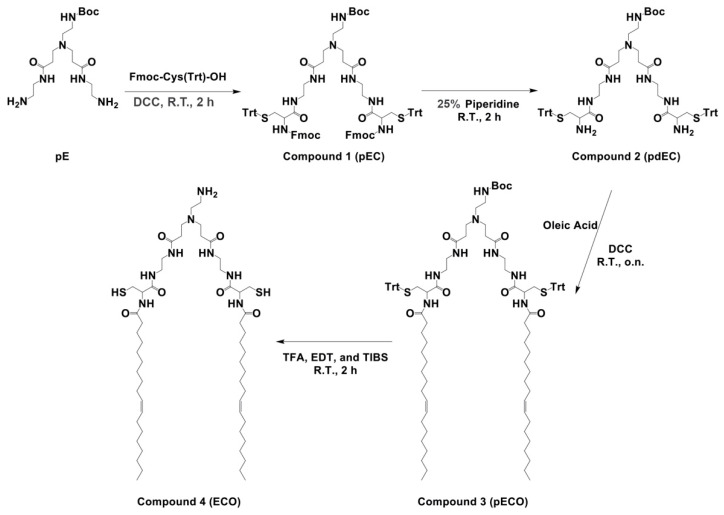
The schematic representation of overall liquid-phase synthetic procedure of the ECO and its intermediates (pEC, pdEC, and pECO).

The liquid-phase chemical synthesis procedure of the amino lipid ECO was designed as shown in Scheme 1. In brief, 1-*N*-(*t*-Boc-amino)ethylimino[bis(2-aminoethyl)propionamide] (pE) was first conjugated with cysteine groups (pEC), followed by deprotection, addition of oleic acid (pECO), and final deprotection to obtain the ECO. Each of the conjugation steps could produce substantial mono-conjugated byproducts due to an incomplete conjugation. These byproducts had similar properties as the fully conjugated products in each step, and were difficult to remove during the purification. To assure complete conjugation and to minimize byproducts of incomplete reactions, excess Fmoc-Cys(Trt)-OH and oleic acid were used in the corresponding steps. The purity and structure of each intermediate were characterized by analytic HPLC and ^1^H-NMR, and MALDI-TOF mass spectrometry. Each intermediate with an acceptable high purity was used for the next step reaction to avoid the generation of impurities that may complicate the purification of the anticipated product. The ^1^H-NMR spectra, HPLC chromatograms, and mass spectra of bis((9H-fluoren-9-yl)methyl)(13-(2-((*tert*-butoxycarbonyl)amino)ethyl)-5,10,16,21-tetraoxo-1,1,1,25,25,25-hexaphenyl-2,24-dithia-6,9,13,17,20-pentaazapentacosane-4,22-diyl)dicarbamate(pEC) and *tert*-butyl (4-amino-13-(3-((2-(2-amino-3-(tritylthio)propanamido)ethyl)amino)-3-oxopropyl)-5,10-dioxo-1,1,1-triphenyl-2-thia-6,9,13-triazapentadecan-15-yl)carbamate (pdEC) are shown in Supplemental Figures S1 and S2. However, after the purification of pEC, there remained excess residual Fmoc-Cys(Trt)-OH (Supplemental Figure S1A). The excess Fmoc-Cys(Trt)-OH residuals did not result in any side products during Fmoc deprotection of pEC. The resulting Cys(Trt)-OH was then able to be removed by washing with a Na_2_CO_3_ aqueous solution.

**Figure 1:**
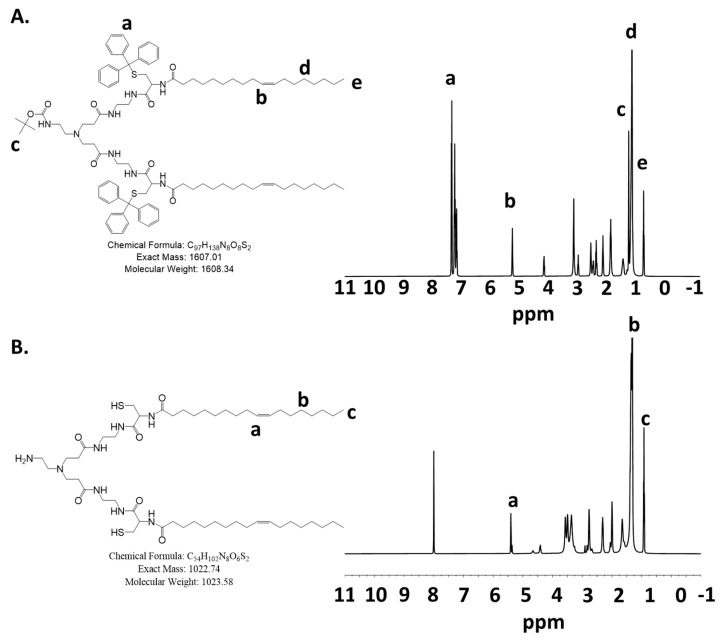
The ^1^H-NMR spectra of (**A**) pECO and (**B**) ECO.

The last deprotection step of pECO to ECO was performed in an ice bath to avoid the oxidation of thiols to form intramolecular disulfide bonds or react with the double bonds in the oleyl groups. The final product ECO was purified by using FLASH chromatography. The fractions containing the ECO were identified by using Ellman’s reagent and confirmed by MALDI-TOF mass spectrometry. The ECO could present as a protonated and unprotonated version and appear in two peaks. The purified ECO was dissolved in water and lyophilized, and then quickly dissolved in ethanol to a 50 mM stock concentration to prevent oxidation of thiols and for nanoparticle formulations with nucleic acids. The final product ECO had a high purity and good solubility in ethanol. Figure 1 shows the ^1^H-NMR spectra of pECO and ECO. The HPLC chromatograms and mass spectra of pECO and ECO are shown in Supplemental Figures S3 and S4.

### 2.2. Formulation and Characterization of ECO/siRNA Nanoparticle

**Figure 2:**
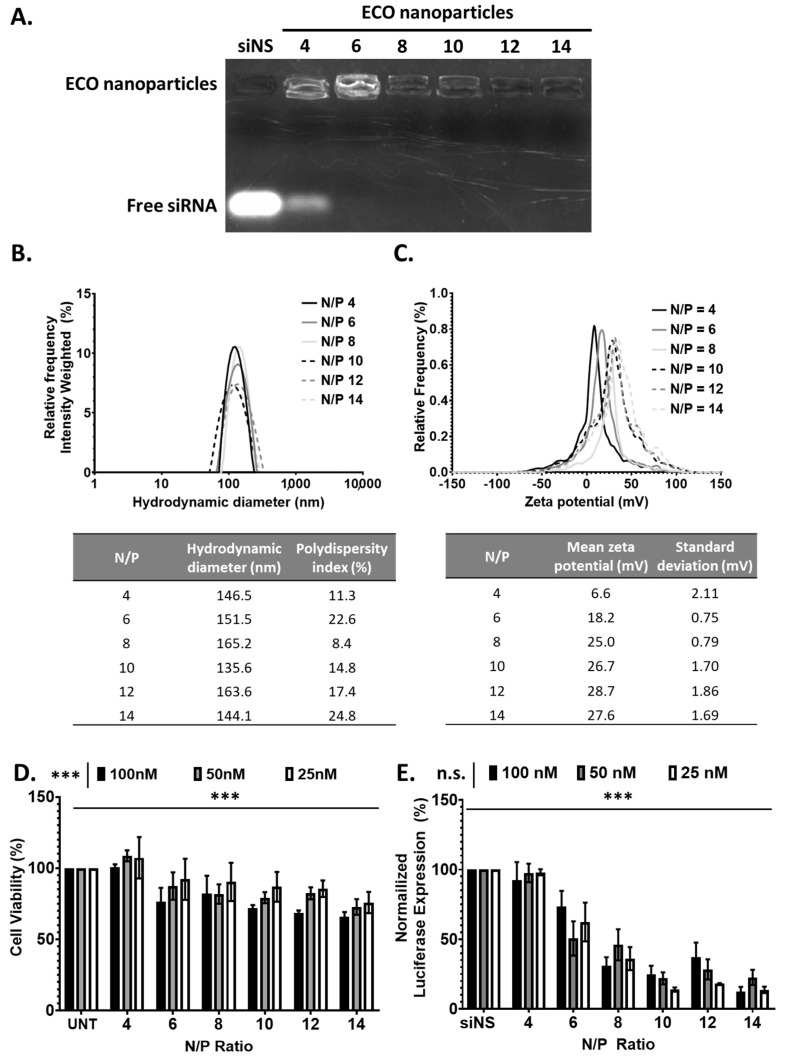
The ECO/siLuc nanoparticle characterization. (**A**) The electrophoresis of ECO/siLuc nanoparticles at various N/P ratios. (**B**,**C**) The hydrodynamic diameter and zeta potential of the ECO/siLuc nanoparticles at N/P ratios 4, 6, 8, 10, 12, and 14. (**D**) ECO/siNS viability at varying N/P ratios after 48 h post transfection. (**E**) Luciferase silencing of ECO/siLuc nanoparticles 48 h post transfection. The bars indicate mean ± s.e.m. *** *p* < 0.005 using two-way ANOVA.

To assess the ability of ECO to form stable ECO/siRNA nanoparticles and to mediate intracellular siRNA delivery for gene silencing, we first evaluated in vitro with siRNA against luciferase (siLuc). ECO/siLuc nanoparticles were formulated through self-assembly in nuclease-free water at N/P ratios of 4–14. The efficiency of siRNA encapsulation with ECO was determined using a gel retardation assay performed with a 1% agarose gel containing ethidium bromide (EtBr). The gel revealed an intense band of free siRNA for the nanoparticles at N/P = 4. The intensity of the free siRNA band decreased significantly and was barely visible at N/P = 6, and undetectable at N/P ≥ 8 (Figure 2A). In addition, the gel revealed that the ethidium bromide staining intensity of the nanoparticles was high at N/P = 4 and 6, and then decreased with increasing N/P ratio. The results indicate the complete encapsulation of siRNA and increased nanoparticle stability at N/P ≥ 8. The nanoparticles were further evaluated for the hydrodynamic diameter and zeta potential using dynamic light scattering. The hydrodynamic diameter of the ECO/siRNA nanoparticles ranged from 135.6 nm to 165.2 nm with no observable trend with the increasing N/P ratio. These variable size distributions may be due to the ECO ability to form a stable nanoparticle with the designated amount of lipid available. The polydispersity index (PDI) of the nanoparticles was in the range of 10–23% for all N/P ratios (Figure 2B). The zeta potential increased with increasing N/P ratios of the nanoparticles with N/P = 4 at 6.6 mV and then increased to approximately 28 mV at N/P of 12 and 14 (Figure 2C). This trend is hypothesized to indicate the level of efficient encapsulation of the siRNA as supported by signal intensity in the agarose gel.

The cytotoxicity of the ECO/siNS nanoparticles was determined at varying N/P ratios and concentrations in GFP-Luc-MDA-MB-231 cells. The cells were transfected for 48 h and viability was measured with CCK8 assay. The cytotoxicity was observed to significantly increase with increasing an siRNA dose; the N/P ratio with an siRNA concentration of 100 nM and N/P 14 had the highest cytotoxicity (Figure 2D). The nanoparticles did not show any cytotoxicity at N/P = 4 at all concentrations. The nanoparticles showed similar low cytotoxicity (~84%) at N/P = 6–12 and 25 and 50 nM siRNA concentration.

The gene silencing efficiency of the ECO/siLuc nanoparticles was evaluated by silencing the reporter gene luciferase in GFP-Luc-MDA-MB-231 breast cancer cells. The cells were transfected in 10% serum media for 48 h at the varying N/P ratios and concentrations (siLuc = 25 nM, 50 nM, 100 nM). The nanoparticles at N/P = 4 did not show any silencing due to poor nanoparticle formulation. Significant gene silencing was observed with an increasing N/P ratio (Figure 2E). At N/P ≥ 10, the nanoparticles showed the highest silencing efficiency compared to the other tested formulations. No significant difference in silencing for all tested concentrations occurred at N/P = 6 and 8 with 25, 50 nM, or 100 nM siRNA concentration. At N/P = 10 and 12, the nanoparticles showed higher silencing efficiency at a low concentration (25 nM). High silencing efficiency at N/P = 14 might be partially attributed to their relatively high cytotoxicity. Taken together, the ECO was able to form nanoparticles with siRNA via self-assembly at the N/P ratio as low as 6 and to efficiently form compact and stable nanoparticles at N/P ratios of 8, 10, and 12. Low cytotoxicity and high gene silencing efficiency were also observed at this N/P ratio range. The ECO/siRNA nanoparticles at N/P = 8, 10, and 12 are promising formulations with high stability, low toxicity, and high gene silencing efficiency for further development.

### 2.3. Formulation and Characterization of ECO/miRNA Nanoparticles

**Figure 3:**
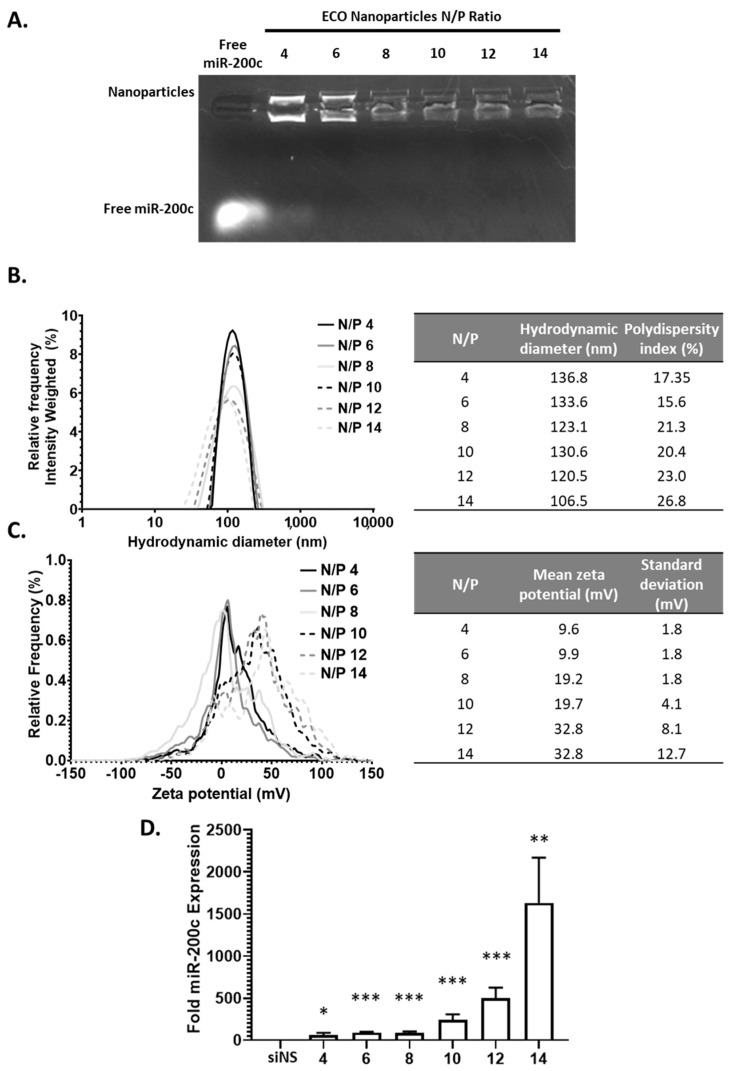
The ECO/miR-200c nanoparticle characterization and transfection. (**A**) The electrophoresis of ECO/miR-200c nanoparticles at N/P ratios 4–14. (**B**,**C**) The hydrodynamic diameter and zeta potential of the ECO/miR-200c nanoparticles. (**D**) The miR-200c levels after 48 h of ECO/miR-200c nanoparticles transfection in MDA-MB-231 cells. The bars indicate mean ± s.e.m. * *p* < 0.05, ** *p* < 0.01, and *** *p* < 0.005 using an unpaired *t*-test.

Next, we explored the ability of the ECO to encapsulate a miRNA (miR-200c) to form stable nanoparticles and their effective delivery to the cytosol to elicit their silencing effect. The ECO/miR-200c nanoparticles were similarly formulated in previous works and at varying N/P ratios [24]. The nanoparticle encapsulation efficiency of miRNA and stability was determined using gel retardation assay similar as before. (Figure 3A) As expected, miR-200c encapsulation revealed a similar trend as compared to siLuc nanoparticles. N/P = 4 revealed a strong ethidium bromide signal of free miR-200c, indicating poor nanoparticle stability, whereas free miR-200c signal decreased at N/P ratio of 6 and was not visible at higher N/P ratios. In addition to the presence of free miR-200c, nanoparticle signal decreased with increasing N/P ratios, further indicating an increase in nanoparticles stability. This together supports the nanoparticle stability of both siRNA and miRNAs at N/P ≥ 8. The ECO/miR-200c nanoparticles exhibited a hydrodynamic diameter range from 106 to 136 nm with a polydispersity of approximately 20% (Figure 3B). These ECO/miR-200c nanoparticles from N/P = 6 to N/P = 12 exhibited an average nanoparticle size decrease from 136 nm to 106 nm increasing with N/P; however, this downward trend was not consistent, which could be explained by the current formulation method as compared to more controlled methods such as microfluidics. Upon measuring the zeta potential of these nanoparticles, they ranged from 0 mV to 20 mV, but did not present a consistent trend with increasing N/P ratios (Figure 3C). The zeta potential increased from N/P 4 to N/P = 14 from 9.6 mV to 32.8 mV. Multiple factors could attribute to these slight alterations. For instance, we hypothesize that due to the method of mixing for nanoparticle formulations, it is not strictly controlled and could contribute to variabilities in nanoparticle size and zeta potential. Another factor that contributes to variabilities is the length of the duplex RNAs, whereas siLuc is smaller than miR-200c duplex.

We next explored the efficacy of the ECO/miR-200c nanoparticles to deliver miR-200c to the cytosol of the MDA-MB-231 cells. GFP-Luc-MDA-MB-231 breast cancer cells were transfected with the nanoparticles at the varying N/P ratios with 100 nM miR-200c in 10% serum media. With increasing N/P ratio, through analysis with qRT-PCR, the amount of miR-200c delivered to the cells increased from 58-fold at N/P = 4 to 1632-fold at N/P = 14 (Figure 3D). This may be a result of the increased stability associated with increased N/P ratio, as we have previously reported the impacts of serum on the nanoparticles and transfection efficiency [22]. Overall, the ECO was capable of making stable nanoparticles with miR-200c for efficient cytosolic delivery at N/P ratios as low as 6.

### 2.4. Formulation and Characterization of ECO/DNA Nanoparticles

**Figure 4:**
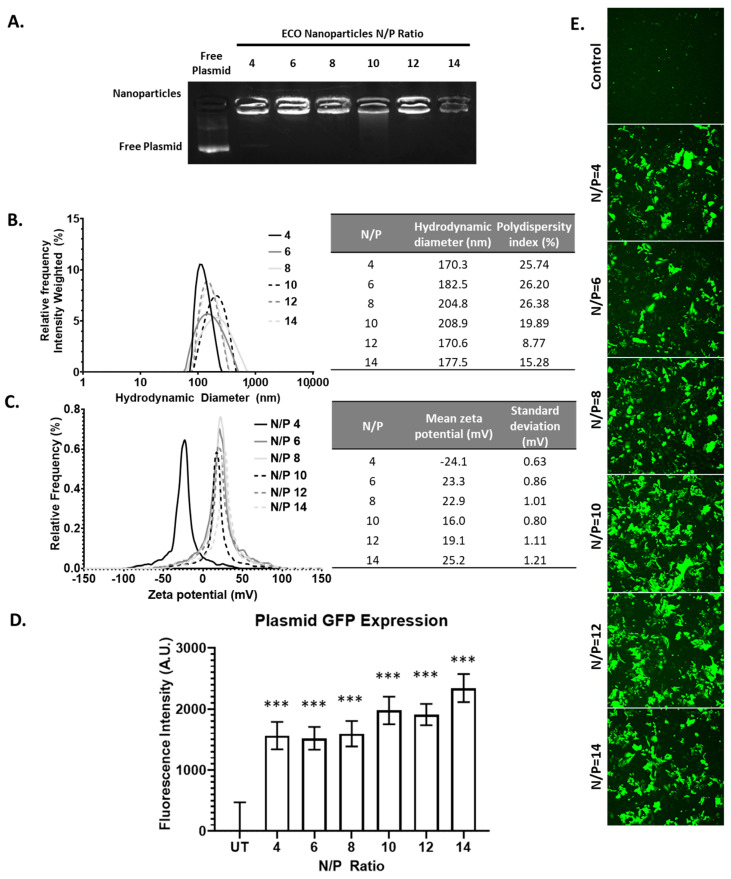
The ECO/pCMV-GFP characterization and transfection. (**A**) The electrophoresis of ECO/pCMV-GFP nanoparticles at N/P ratios 4–14. (**B**,**C**) The hydrodynamic diameter and zeta potential of the ECO/pCMV-GFP nanoparticles at various N/P ratios. (**D**,**E**) The GFP fluorescence intensity after 48 h of ECO/pCMV-GFP nanoparticles transfection in ARPE-19 cells. The bars indicate mean ± s.e.m. *** *p* < 0.005 using an unpaired *t*-test.

Finally, we characterized the ECO formulation using a plasmid DNA encoding GFP reporter gene (*pCMV-GFP*). The overall plasmid encapsulation efficiency of the plasmid with the ECO was evaluated through gel retardation assay. Across all N/P ratios of the ECO/*pCMV-GFP* nanoparticles, no ethidium bromide signal was observed for free plasmid. Compared to siRNA and miRNA formulations, characterization of nanoparticles revealed no trends with increasing N/P ratios, including the signal intensity (Figure 4A). These characteristics may indicate that the ECO and large plasmid DNA form tightly packed nanoparticles even at low N/P ratios as compared to the smaller counterparts, siRNA and miRNA. Upon further characterization with DLS, the ECO/*pCMV-GFP* nanoparticles’ hydrodynamic diameter ranged from 170 nm to 200 nm (Figure 4B). Ratios from N/P = 4 to N/P = 10 revealed an increasing trend in nanoparticle size from 170 nm to 209 nm; however, N/P = 12 and 14 resulted in smaller nanoparticles (ca. 170 nm). The results indicate that more compact nanoparticles might be formed with excess ECO. The zeta potential of these plasmid nanoparticles followed a similar trend to siRNA, where the overall zeta potential ranged from −25 mV to 25 mV (Figure 4C). N/P = 4 nanoparticles displayed a −24.1 mV zeta potential, while N/P ≥ 6 expressed a positive zeta potential.

We transfected ARPE-19 cells with the ECO/pCMV-GFP (pCMV-GFP = 1 μg/mL) nanoparticles at various N/P ratios for 4 h. After 48 h, we assessed the overall GFP expression signal across all N/P ratios as compared to the untransfected control through confocal microscopy (Figure 4D,E). We then quantified this signal for quantitative assessment. Here, we observed the overall GFP expression increasing with higher N/P ratios, with N/P = 14 being the highest. Taken all together, however, plasmids delivered with the ECO could be utilized at lower N/P ratios, as nanoparticles appear stable and provide significant GFP signal upon transfection.

## 3. Materials and Methods

All reactions were carried out in anhydrous solvents. All reagents and anhydrous solvents were commercially available without the need for further purification. Dichloromethane (DCM), dimethylformamide (DMF), methanol, ethyl acetate, and tetrahydrofuran (THF) were obtained from Fisher Scientific. Anhydrous *N*,*N*-diisopropylethylamine (DIPEA) was purchased from Alfa Aesar (Ward Hill, MA, USA). Trifluoroacetic acid (TFA) was purchased from Oakwood Products, Inc. (West Columbia, SC, USA). *Tert*-butyl (2-(bis(3-((2-aminoethyl)amino)-3-oxopropyl)amino)ethyl)carbamate (pEC) was purchased from Alichem (San Diego, CA, USA). The Fmoc-Cyt(Trt)-OH used in the chemical synthesis was purchased from Novabiochem (Darmstadt, Germany). *N*,*N*′-Dicyclohexylcarbodiimide (DCC), piperidine, 1,2-ethanedithiol (EDT), triisobutylsilane (TIBS), oleic acid, and *N*-(3-(dimethylamino)propyl)-*N′*-ethylcarbodiimide hydrochloride (EDC) were purchased from the Sigma-Aldrich Corporation (St. Louis, MO, USA). 5,5′-Dithiobis-(2-nitrobenzoic acid) (DTNB) was purchased from Pierce Inc. (Rockford, IL, USA).

### 3.1. Cell Lines and Reagents

MDA-MB-231 and ARPE-19 cell lines were purchased from ATCC (Manassas, VA, USA). MDA-MB-231 and ARPE-19 cells were maintained in Dulbecco’s Modified Eagle’s Medium (DMEM, Gibco, Grand Isle, VT, USA) supplemented with 10% fetal bovine serum (FBS, Gibco) and 1% Penicillin/Streptomycin. The cells were cultured in a humidified incubator at 37 °C with 5% CO_2_. The MDA-MB-231 cells were also engineered to express firefly luciferase and GFP with a lentivirus CMV-Luciferase (Firefly)-2A-GFP (Neo) from Amsbio (Cambridge, MA, USA) and sorted for selection using flow cytometry (GFP-Luc-MDA-MB-231). The siRNA for luciferase (siLuc) was purchased from Dharmacon (Lafayette, CO, USA) (sense: 5′-CCUACGCCGAGUACUUCGA-3′; antisense, 5′-GGAUGCGGCUCAUGAAGCU-3′). The miR-200c duplex (sense: 5′-UAAUACUGCCGGGUAAUGAUGGA-3′; antisense: 5′-UCCAUCAUUACCCGG CAGUAUUA-3′) was purchased from Integrated DNA Technologies (Coralville, IA, USA).

### 3.2. HPLC Conditions

The conditions for analytical HPLC: HPLC solutions were solvent A [1% TFA in water] and solvent B [acetonitrile (ACN)]. Column: Gemini 5 μm C18 110 Å LC Column 250 × 4.6 mm. A 10 min isocratic at 0% B was used, from 10 to 30 min a linear gradient from 0% to 100 %B, followed by isocratic at 100% B from 30 to 55 min at a 1 mL/min flow rate using UV detection at 256 nm.

### 3.3. Synthesis of Compound ***1*** (pEC)

In a round bottom flask, *N*,*N*′-Dicyclohexylcarbodiimide (DCC) (2.0 g, 6.1 mmol) and Fmoc-Cys(Trt)-OH (7.5 g, 12.9 mmol) were dissolved in 40 mL of THF. In a separate flask, *tert*-butyl (2-(bis(3-((2-aminoethyl)amino)-3-oxopropyl)amino)ethyl)carbamate (pE) (1 g, 2.6 mmol) was dissolved in 20 mL of THF and added dropwise to the reaction flask. The solution was allowed to react for 2 h at RT, and the DCU precipitate was removed by filtration. The THF was removed by rotary evaporation under a vacuum until completely dry. The DCM (60 mL) was added to the crude product and poured into a separatory funnel. Sixty mL of 5% Na_2_CO_3_ aqueous solution was added to wash the DCM layer 3 times. The DCM fraction was collected by phase separation and was dried using sodium sulfate and filtered with grade 2 V folded qualitative filter papers (Cytiva, Marlborough, MA, USA). The DCM was removed by rotary evaporation under a vacuum to dryness. The residue was dissolved in 5 mL of DCM, and solids were removed. The product was then precipitated in excess diethyl ether and allowed to dry to obtain the final product bis((9H-fluoren-9-yl)methyl) (13-(2-((*tert*-butoxycarbonyl)amino)ethyl)-5,10,16,21-tetraoxo-1,1,1,25,25,25-hexaphenyl-2,24-dithia-6,9,13,17,20-pentaazapentacosane-4,22-diyl)dicarbamate (pEC, 3.0 g, 76%). ^1^H-NMR (500 MHz, MeOD) δ 7.54 (16H, C=CH), 7.18 (d, J = 7.6, 12H, CH), 7.07 (t, J = 7.7, 12H, CH), 6.95 (t, J = 7.3, 6H, CH), 4.42 (q, J = 9.8, 2H, HC-CON), 4.31 (d, J = 9.1, 4H, HC-OOC), 4.15 (t, J = 9.7, 2H, CH), 3.26 (12H, N-CH, CON-CH), 3.12 (t, J = 6.5, 2H, N-CH), 2.69 (t, J = 6.5, 4H, HC-S), 2.61 (q, J = 6, 2H, CON-CH), 2.31 (t, J = 6.7, 4H, HC-CON), 2.00 (q, J = 6.7, 8H, HC-C=C), 1.65 (p, J = 7.0, 4H, CH_2_-C), 1.41 (s, 9H, CH_3_-C). MALDI-TOF MS m/z calculated for C_91_H_94_N_8_O_10_S_2_ (M + 1) 1523.92, found m/z 1523.64; calculated for C_91_H_94_N_8_O_10_S_2_ Na (M + Na) 1545.92, found m/z 1546.67; calculated for C_72_H_80_N_8_O_10_S_2_ of one less trityl group (M + 1) 1281.60, found 1281.56; calculated for C_72_H_80_N_8_O_10_S_2_ Na (M + Na) 1303.56, found m/z 1304.59.

### 3.4. Synthesis of Compound ***2*** (pdEC)

The pEC (3.0 g, 2.0 mmol) was deprotected in 100 mL of DMF with 25% piperidine for 2 h at RT with stirring. The solvent was removed by rotary evaporation under a vacuum to dryness. The residue was redissolved in 30 mL of DCM, and washed with 5% Na_2_CO_3_ aqueous solution (60 mL) 2 times. The DCM layer was collected and dried using dried sodium sulfate, and then filtered. The solvent was removed by rotary evaporation under a vacuum. The residue was redissolved in 5 DCM and added to 50 mL hexanes to give a colorless precipitate. The purification process was repeated one more time to give a colorless solid, *tert*-butyl (4-amino-13-(3-((2-(2-amino-3-(tritylthio)propanamido)ethyl)amino)-3-oxopropyl)-5,10-dioxo-1,1,1-triphenyl-2-thia-6,9,13-triazapentadecan-15-yl)carbamate (pdEC, 2.0 g, 91%). ^1^H-NMR (500 MHz, MeOD) δ 7.41 (d, J = 7.6, 12H, CH), 7.31 (t, J = 7.7, 12H, CH), 7.23 (t, J = 7.3, 6H, CH), 4.61 (p, J = 7.1, 2H, HC-N), 3.3 (12H, N-CH, CON-CH), 3.12 (t, J = 6.5, 2H, N-CH), 2.69 (t, J = 6.5, 4H, HC-S), 2.60 (q, J = 6, 2H, 2.50 (t, J = 8.0, 4H, HC-CON), 2.02 (q, J = 6.7, 8H, HC-C=C), 1.60 (p, J = 7.0, 4H, CH_2_-C), 1.42 (s, 9H, CH_3_-C). MALDI-TOF MS m/z calculated for C_61_H_74_N_8_O_6_S_2_ (M + 1) 1079.43, found m/z 1080.72; calculated for C_61_H_74_N_8_O_6_S_2_ Na (M + Na) 1102.4, found m/z 1101.70.

### 3.5. Synthesis of Compound ***3*** (pECO)

To synthesize *tert*-butyl (*(Z)*-3-(3-((2-(2-(*(Z)*-octadec-10-enamido)-3-(tritylthio)propanamido)ethyl)amino)-3-oxopropyl)-6,11,14-trioxo-12-((tritylthio)methyl)-3,7,10,13-tetraazahentriacont-23-en-1-yl)carbamate (pECO), pdEC (2.0 g, 1.8 mmol) and *N*,*N*’-Dicyclohexylcarbodiimide (DCC) (2.0 g, 6.1 mmol) were dissolved in 100 mL of ethyl acetate. To the solution, 2.0 mL of oleic acid (excess) was added dropwise, and was allowed to react 2 h at RT with stirring before the DCU precipitate was removed. The ethyl acetate was removed by rotary evaporation at 50 mbar, and the residue was redissolved in the DCM. The product was purified by FLASH chromatography with a silica gel column using a DCM:methanol gradient. The final product was concentrated and dried to a yellowish solid (1.5 g, 52%). ^1^H-NMR (500 MHz, MeOD) δ 7.39 (d, J = 7.6, 12H, CH), 7.29 (t, J = 7.7, 12H, CH), 7.22 (t, J = 7.3, 6H, CH), 5.34 (q, J = 7.2, 4H, CH=C), 4.27 (q, J = 7.1, 2H, CON-CH), 3.26 (12H, N-CH, CON-CH), 3.12 (t, J = 6.5, 2H, N-CH), 2.69 (t, J = 6.5, 4H, HC-S), 2.61 (q, J = 6, 2H, CON-CH), 2.28 (t, J = 6.3, 4H, HC-CON), 2.02 (q, J = 6.7, 8H, HC-C=C), 1.60 (p, J = 7.0, 4H, CH_2_-C), 1.41 (s, 9H, CH3-C), 1.31 (t, J = 6.8, CH_2_-C), 0.91 (t, J = 6.7, 6H, CH_3_-C). MALDI-TOF MS m/z calculated for C_97_H_138_N_8_O_8_S_2_ (M + 1) 1608.34, found m/z 1609.40; calculated for C_97_H_138_N_8_O_8_S_2_ Na (M + Na) 1631.33, found m/z 1630.36.

### 3.6. Synthesis of Compound ***4*** (ECO)

The pECO (1.5 g, 0.93 mmol) was dissolved in 15 mL of DCM:TFA:TIBS:EDT (20:67:10:3.3) and allowed to react for 2 h in an ice bath with stirring. The solvents were removed via rotary evaporation under a vacuum, and the residue was redissolved in 10 mL of DCM then purified using FLASH chromatography with a silica gel column with a 1% TFA/DCM:1% TFA/methanol gradient. The resulting *N*,*N*’-(11-(2-aminoethyl)-1,21-dimercapto-3,8,14,19-tetraoxo-4,7,11,15,18-pentaazahenicosane-2,20-diyl)bis(octadec-10-enamide) (ECO) was confirmed with Elman’s reagent and dried to produce an oily product (300 mg, 31%). Once confirmed, the ECO was redissolved in ethanol to make a 50 mM ECO stock solution for nanoparticle formation. ^1^H-NMR (500 MHz, MeOD) δ 5.40 (q, J = 7.2, 4H, CH=C), 4.42 (q, J = 7.1, 2H, CON-CH), 3.38 (12H, N-CH, CON-CH), 3.26 (t, J = 6.5, 2H, N-CH), 2.69 (t, J = 6.5, 4H, HC-S), 2.61 (q, J = 6, 2H, CON-CH), 2.31 (t, J = 6.3, 4H, HC-CON), 2.00 (q, J = 6.7, 8H, HC-C=C), 1.65 (p, J = 7.0, 4H, CH_2_-C), 1.31 (t, J = 6.8, CH_2_-C), 0.92 (t, J = 6.7, 6H, CH_3_-C). MALDI-TOF MS m/z calculated for C_54_H_103_N_8_O_6_S_2_ (M + 1) 1024.59, found m/z 1024.02; calculated for C_54_H_102_N_8_O_6_S_2_Na (M + Na) 1046.57, found m/z 1045.05.

### 3.7. ECO Nanoparticle Formulation

The ECO/nucleic acid nanoparticles were prepared as previously described [22,23]. In short, the ECO nanoparticles were prepared at various N/P ratios between 4 and 14. The siRNA, miR-200c, and plasmid DNA were diluted into nuclease-free water from a 25 μM stock, and the ECO was added from a 5 mM stock solution in ethanol. The solution was briefly mixed and then vortexed for 30 min at room temperature.

### 3.8. Nanoparticle Characterization

The characterization of the ECO/nucleic acid nanoparticles was conducted as previously described [20,21,22,23]. In brief, nanoparticles were diluted into NF water (1:20) (pH = 5.5). Dynamic light scattering was conducted using the Litesizer 500 from Anton Paar GmbH (Graz, Austria) at 25 °C to hydrodynamic diameter, polydispersity index, and zeta potential. Agarose gel electrophoresis was conducted as previously described [21]. In brief, 20 µL of nanoparticle formulation was mixed with 4 µL of 6X loading dye (Roche, Basel, Switzerland) and loaded onto a 1% agarose gel containing ethidium bromide. The gel was submerged in 0.5× Tris/Borate/ethylenediaminetetraacetic acid and ran with 100 V for 15 min. The free siRNA was prepared similarly, whereas the equivalent amount of genetic material was mixed with 4 µL of loading dye. The bands were visualized using the ChemiDoc XRS system from BioRad (Hercules, CA, USA).

### 3.9. In Vitro Cell Viability

GFP-Luc-MDA-MB-231 cells were seeded onto a 96-well plate at 10,000 cells/well and then treated with the ECO/siRNA nanoparticles (N/P = 4, 6, 8, 10, 12, and 14, siRNA conc. = 25 nM, 50 nM, or 100 nM) for 48 h. After transfection, cells were washed with PBS and 100 µL of fresh media was added with the addition of 10 µL of CCK8 reagent (Dojindo, Rockville, MD, USA). The cells were allowed to incubate at 37 °C for 3 h until read with a SpectraMax microplate reader (Molecular Devices, Sunnyvale, CA, USA). The absorbance was measured at 450 nm after incubation. The normalized absorbance was normalized to untransfected control.

### 3.10. In Vitro Luciferase Silencing Efficiency

GFP-Luc-MDA-MB-231 cells were seeded onto a 12-well plate at 3.0 × 10^5^ cells/well and treated with the ECO/siLuc nanoparticles (N/P = 4, 6, 8, 10, 12, and 14, siRNA conc. = 25 nM, 50 nM, or 100 nM) for 48 h. Following transfection, the cells were washed with PBS, and 200 µL of reporter lysis buffer (Promega, Madison, WI, USA) was added. The cells were frozen at -80 °C overnight and then thawed. The samples were 10,000× *g* for 5 min, and 20 µL of cell lysates were added to a 96-well plate in triplicate. Utilizing the Promega luciferase kit, 100 µL of Luciferase Assay Reagent was added to each well, and the luminescence was measured for 10 s using a SpectraMax microplate reader (Molecular Devices, Sunnyvale, CA, USA). The luminescence was normalized to total protein content using BCA assay (Thermo Scientific, Waltham, MA, USA). The normalized luminescence was normalized to untransfected control.

### 3.11. Semi-Quantitative RT-PCR Analyses

Semiquantitative real-time PCR was conducted as described previously [24]. Briefly, MDA-MB-231 had total RNA, including miRNAs, extracted using the RNeasy Plus Mini Kit from Qiagen (Germantown, MD, USA) as per the manufacturer’s instructions. The RNA was converted to cDNA through reverse transcription using the miScript II RT Kit (Qiagen, Hilden, Germany). qPCR was performed using a miScript SYBR Green PCR kit (Qiagen), containing human RNU6B (RNU6-2) miScript Primer Assay and miScript Universal Primer. miRNA expression levels were normalized to U6 (Qiagen). The primer sequences used were miR-200c: Fwd 5′-TAATACTGCCGGGTAATGATGGA-3′ and Rev miScript Universal Primer (Qiagen).

### 3.12. GFP Fluorescence Measuring

ARPE-19 cells were seeded onto a 96-well plate at 10,000 cells/well and then treated with the ECO/pCMV-GFP nanoparticles (pCMV-GFP = 1 μg/mL) at N/P ratios of 4, 6, 8, 10, 12, and 14 for 4 h. The cells were then washed with PBS, and fresh media was added for the remaining time. After 48 h, the fluorescence was measured using a SpectraMax microplate reader (Excitation 488 nm, emission 509 nm). The control fluorescence was subtracted from treatment groups for normalization. The qualitative assessment of the GFP expression was evaluated using confocal microscopy. ARPE-19 cells were seeded onto a 12-well plate at 10 × 10^5^ cells/well and allowed to adhere overnight. The cells were then transfected as stated before, and washed with PBS after 4 h then replaced with fresh media. The cells were imaged using an Olympus FV1000 confocal microscope.

### 3.13. Statistical Analysis

All experiments were conducted at least 3 times. Statistical significance was conducted using Graphpad software, with statistical significance between two groups calculated using an unpaired t-test or two-way ANOVA. The data is represented as mean ± s.e.m.

## 4. Conclusions

Non-viral vectors are currently the most common method used to administer genetic materials, and are being utilized in vaccines and treatments in vivo. The amino lipid ECO is a proven effective gene delivery vehicle for various diseases with minimal toxicity; however, solid-phase synthesis has poor scalability. Thus, we have reported a convenient synthesis in liquid-phase for the production of the ECO with high purity. We characterized each intermediate compound and final product to determine purity, and tested the ECO lipid to ensure its functionality as a gene delivery system.

## Data Availability

Data is contained within the article and supplementary material.

## Supplementary Materials

The following are available online at https://www.mdpi.com/article/10.3390/ph14101016/s1, Figure S1: Characterization of pEC, Figure S2: Characterization of pdEC, Figure S3: Characterization of pECO, Figure S4: Characterization of ECO.
