# Correction: Schilb et al. Optimization of Synthesis of the Amino Lipid ECO for Effective Delivery of Nucleic Acids. *Pharmaceuticals* **2021**, *14*, 1016

**DOI:** 10.3390/ph18111594

**Published:** 2025-10-22

**Authors:** Andrew L. Schilb, Josef H. Scheidt, Amita M. Vaidya, Zhanhu Sun, Da Sun, Sangjoon Lee, Zheng-Rong Lu

**Affiliations:** 1 Department of Biomedical Engineering, School of Engineering, Case Western Reserve University, Cleveland, OH 44106, USA; als241@case.edu (A.L.S.); jhs192@case.edu (J.H.S.); amv74@case.edu (A.M.V.); sunzhanhu@hotmail.com (Z.S.); dxs564@case.edu (D.S.); sxl1755@case.edu (S.L.); 2 Case Comprehensive Cancer Center, Case Western Reserve University, Cleveland, OH 44106, USA

## Text Correction

There was an error in the original publication [1], specifically in the Materials and Methods Section, Section 3.6. Synthesis of Compound **4** (ECO). The sentence originally stated the following:

“MALDI-TOF MS m/z calculated for C_97_H_138_N_8_O_8_S_2_ (M + 1) 1608.34, found m/z 1609.40; calculated for C_97_H_138_N_8_O_8_S_2_ Na (M + Na) 1631.33, found m/z 1630.36.”

This has to be corrected to the following:

“MALDI-TOF MS m/z calculated for C_54_H_103_N_8_O_6_S_2_ (M + 1) 1024.59, found m/z 1024.02; calculated for C_54_H_102_N_8_O_6_S_2_Na (M + Na) 1046.57, found m/z 1045.05.”

The authors state that the scientific conclusions are unaffected. The Academic Editor approved this correction. The original publication has also been updated.
