# Supplementary material for: Optimization of Synthesis of the Amino Lipid ECO for Effective Delivery of Nucleic Acids
Source: Pharmaceuticals (Basel). 2021 Oct 2;14(10):1016. doi: 10.3390/ph14101016 (PMC8537419; doi:10.3390/ph14101016)
Supplement: Supplementary file 1 [file pharmaceuticals-14-01016-s001.zip › pharmaceuticals-1365220-supplementary.pdf]

Supplemental Figures:

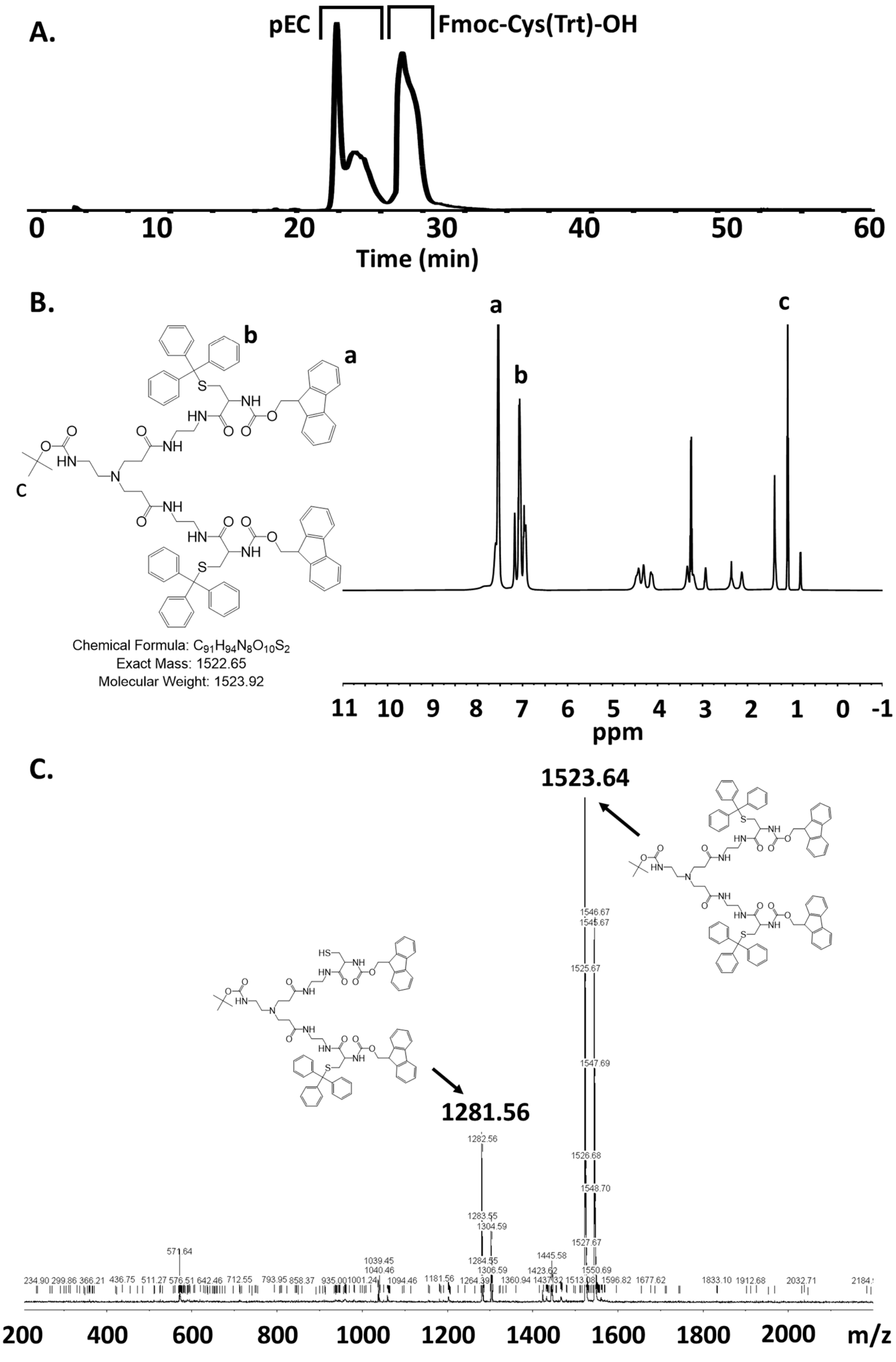

Figure S1: Characterization of pEC. **A**) HPLC analysis at 256nm **B**) ^{1}H-NMR, and **C**) MALDI-TOF mass spectrum of pEC revealing the presence of Fmoc-Cys(Trt)-OH in addition to pEC.

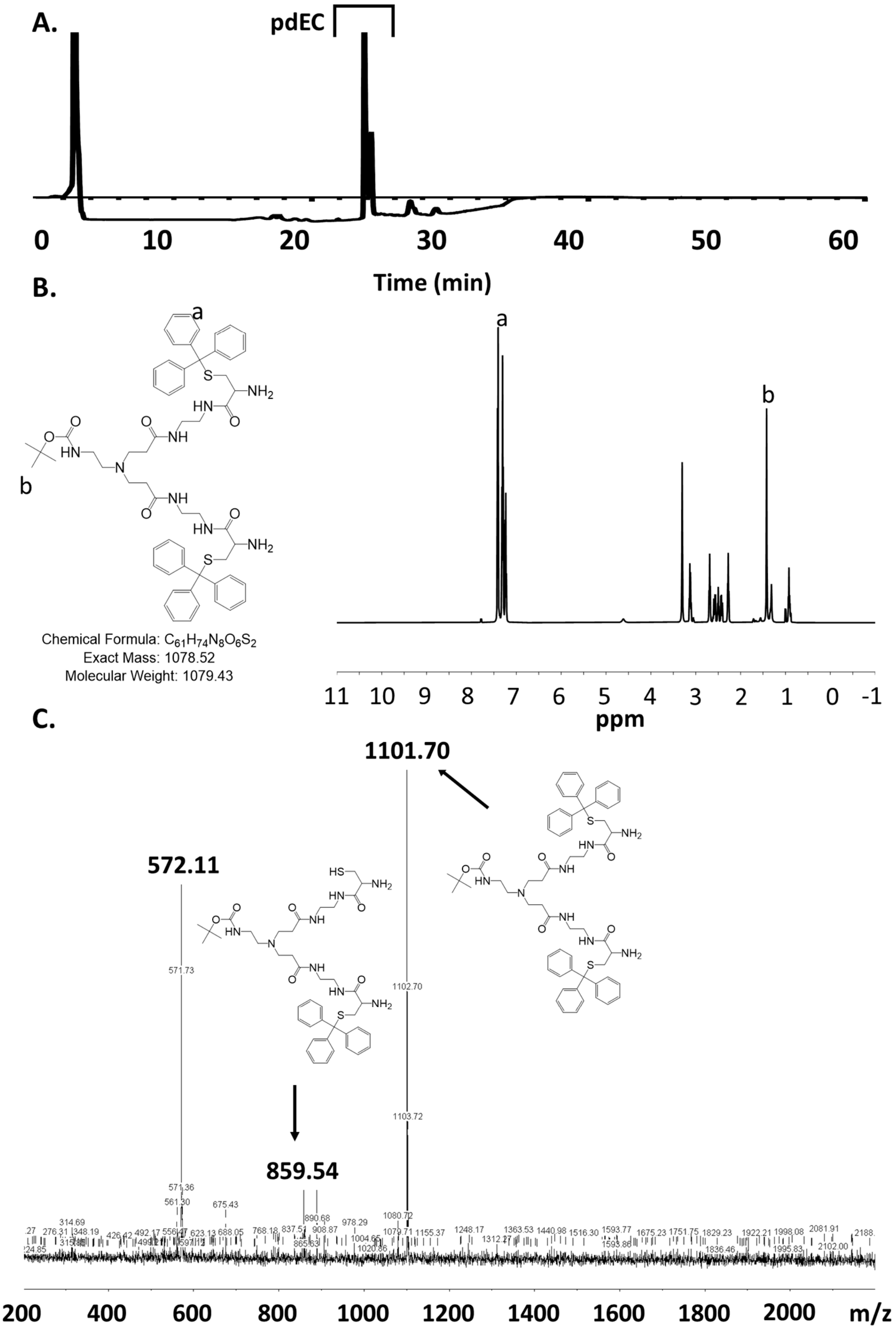

Figure S2. Characterization of pdEC. **A**) HPLC at 256nm analysis **B**) ^{1}H-NMR, and **C**) MALDI-TOF mass spectrum of pdEC revealing strong pdEC signal with no indication of impurities.

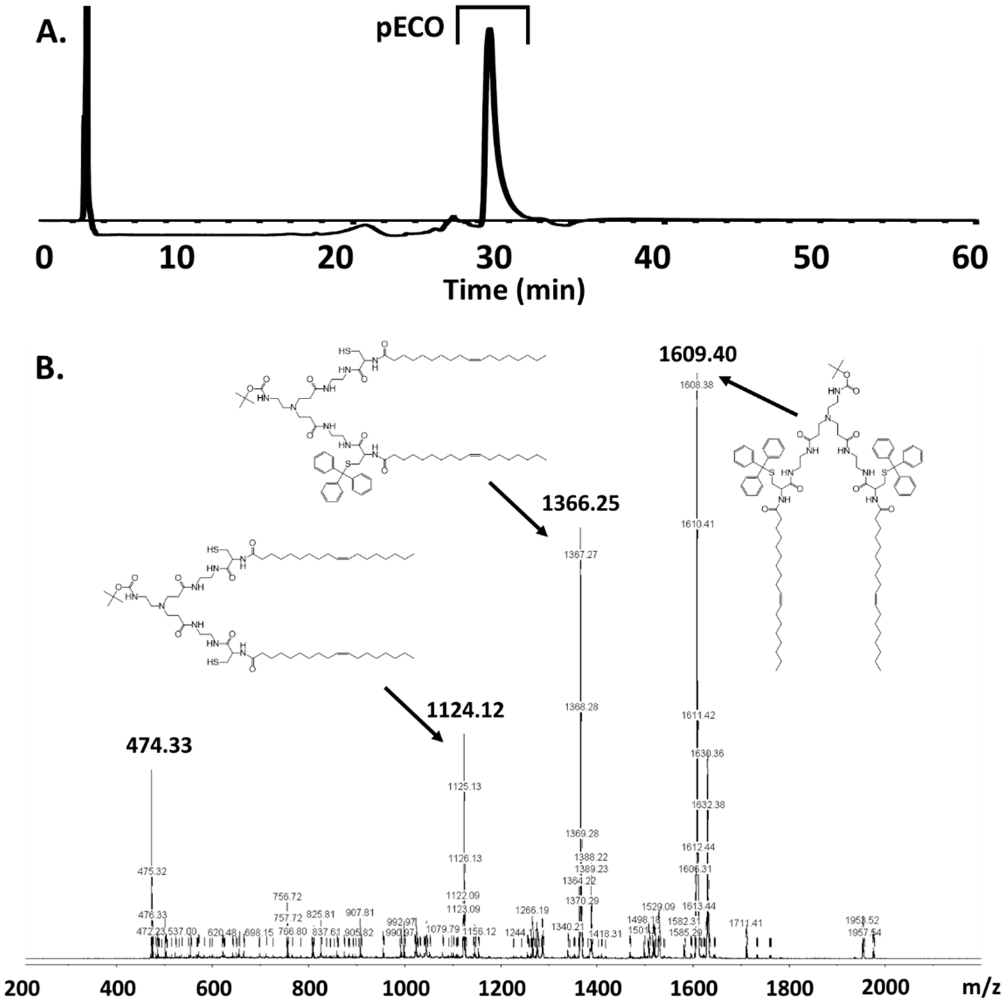

Figure S3. Characterization of pECO. **A**) HPLC analysis at 256nm and **B**) MALDI-TOF mass spectrum of pECO showing strong presence of pECO with various partially deprotected pECO.

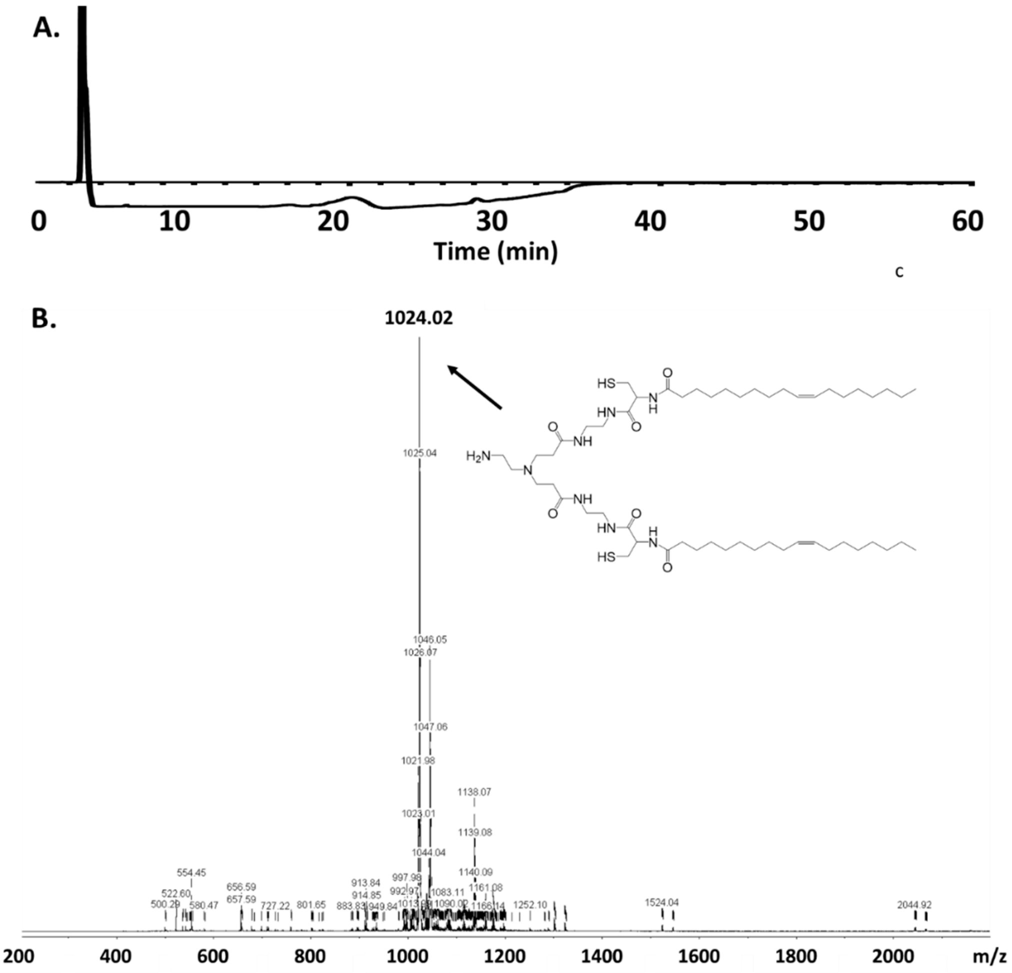

Figure S4. Characterization of ECO. **A**) HPLC analysis at 256nm and **B**) MALDI-TOF mass spectrum of ECO revealing successful removal of trityl cations and pure ECO.
